# Effects of ICI 182,780, an ERα and ERβ antagonist, and G-1, a GPER agonist, on autophagy in breast cancer cells

**DOI:** 10.31744/einstein_journal/2020AO4560

**Published:** 2020-04-13

**Authors:** Mari Luminosa Muler, Fernanda Antunes, Gabriel Cicolin Guarache, Rafaela Brito Oliveira, Rodrigo Portes Ureshino, Claudia Bincoletto, Gustavo José da Silva Pereira, Soraya Soubhi Smaili

**Affiliations:** 1 Departamento de Farmacologia Escola Paulista de Medicina Universidade Federal de São Paulo São PauloSP Brazil Departamento de Farmacologia , Escola Paulista de Medicina , Universidade Federal de São Paulo , São Paulo , SP , Brazil .; 2 Departamento de Ciências Biológicas Universidade Federal de São Paulo DiademaSP Brazil Departamento de Ciências Biológicas , Universidade Federal de São Paulo , Diadema , SP , Brazil .

**Keywords:** Autophagy, Breast neoplasms, Estrogens, Receptors, estrogen, Receptors, G-protein-coupled, Fulvestrant; Cellular proliferation, MCF-7 Cells

## Abstract

**Objective:**

To investigate if ICI 182,780 (fulvestrant), a selective estrogen receptor alpha/beta (ERα/ERβ) antagonist, and G-1, a selective G-protein-coupled receptor (GPER) agonist, can potentially induce autophagy in breast cancer cell lines MCF-7 and SKBr3, and how G-1 affects cell viability.

**Methods:**

Cell viability in MCF-7 and SKBr3 cells was assessed by the MTT assay. To investigate the autophagy flux, MCF-7 cells were transfected with GFP-LC3, a marker of autophagosomes, and analyzed by real-time fluorescence microscopy. MCF-7 and SKBr3 cells were incubated with acridine orange for staining of acidic vesicular organelles and analyzed by flow cytometry as an indicator of autophagy.

**Results:**

Regarding cell viability in MCF-7 cells, ICI 182,780 and rapamycin, after 48 hours, led to decreased cell proliferation whereas G-1 did not change viability over the same period. The data showed that neither ICI 182,780 nor G-1 led to increased GFP-LC3 puncta in MCF-7 cells over the 4-hour observation period. The cytometry assay showed that ICI 182,780 led to a higher number of acidic vesicular organelles in MCF-7 cells. G-1, in turn, did not have this effect in any of the cell lines. In contrast, ICI 182,780 and G-1 did not decrease cell viability of SKBr3 cells or induce formation of acidic vesicular organelles, which corresponds to the final step of the autophagy process in this cell line.

**Conclusion:**

The effect of ICI 182,780 on increasing acidic vesicular organelles in estrogen receptor-positive breast cancer cells appears to be associated with its inhibitory effect on estrogen receptors, and GPER does notseem to be involved. Understanding these mechanisms may guide further investigations of these receptors’ involvement in cellular processes of breast cancer resistance.

## INTRODUCTION

Breast cancer is one major cause of death among women, in Brazil and other countries. ^1^ Treatment can be curative in some cases, and one of the most commonly used protocol involves surgical excision, followed by chemotherapy/radiation therapy. In case of hormone-sensitive breast cancer, ^2^ hormone therapy consists of inhibiting estrogen synthesis or interfering in signaling mediated by this steroid. ^3^ In these cases, the standard treatment uses tamoxifen, a selective estrogen receptor (ER) modulator, as well as ICI 182,780 (fulvestrant − herein referred to as ICI) and aromatase inhibitors, ^4^ to reduce estrogen stimulation and the related protein synthesis and cell proliferation.

The first ERs described were alpha (ERα) and beta (ERβ), the primary mechanism of which is their action as transcription factors. Later, a G-protein-coupled ER (GPER) was described, with seven G-protein-coupled transmembrane domains, the expression pattern of which can reveal the aggressiveness of the tumor when associated with ER expression. ^5^ Even though ICI is an antagonist of classic receptors (ERα and ERβ), it is a GPER agonist, ^5^ and the related signaling pathways are still not fully understood. Evidence points to ERα expression as a potential modulator of cancer cell response to different therapies, by means of autophagy. ^6 , 7^ Macroautophagy, herein referred to as “autophagy”, is a lysosomal pathway for recycling of macromolecules and cell organelles sequestered into autophagosomes, bounded by a double membrane. They merge with lysosomes to form autolysosomes, within which degradation takes place. ^8^

Studies showed that activating autophagy in normal cells can prevent tumor formation, and inhibiting autophagy could be beneficial in established tumors. On the other hand, cytotoxic drugs used in cancer treatment are known to induce autophagy and could trigger cell death in apoptosis-deficient cells. ^9^ Since autophagy has a double role in tumorigenesis and tumor progression, understanding these mechanisms could favor the discovery of potential treatment targets in breast cancer.

## OBJECTIVE

To assess if ICI 182,780 (fulvestrant), a selective antagonist of estrogen receptors alpha and beta (ERα/ERβ), and G-1, a selective G-protein-coupled estrogen receptor (GPER) agonist, can potentially induce autophagy in breast cancer cell lines MCF-7 and SKBr3, and if G-1 can affect cell viability.

## METHODS

This study was approved by the Ethics Committee of the *Universidade Federal de São Paulo* under opinion number 1748/10.

### Reagents

DMEM/F12, fetal bovine serum (FBS), penicillin/streptomycin and trypsin/ethylenediaminetetraacetic acid (EDTA) 0.5% were obtained from Invitrogen™ of Brazil (St. Louis, MO, USA). ICI 182,780 (AstraZeneca of Brazil; Cotia, São Paulo, Brazil), 1-[4-(6-bromobenzo[1,3] dioxol-5yl)-3a,4,5,9b-tetrahydro-3H-cyclopenta[c]quinolin-8-yl]-ethanone (G-1; Calbiochem ^®^ ; Merck Biosciences, Darmstadt, Germany), 17β-estradiol 3-benzoate (17β-estradiol, E2) (Sigma Chemical Co.; St Louis, MO, USA) and 4,4’,4’’-(4-propyl-(1H)-pyrazole-1,3,5-triyl) trisphenol (PPT). Rapamycin (RAP) and 3-(4,5-dimethylthiazol-2-yl)-2,5-diphenyltetrazolium bromide (MTT) were obtained from Sigma Aldrich (St. Louis, MO, USA). Acridine orange (AO) was obtained from Molecular Probes (Eugene, OR, USA). The GFP-LC3 plasmid was obtained from the National Institute for Infectious Diseases, USA, and *Istituto di Ricovero e Cura a Carattere Scientifico L. Spallanzani* , Rome, Italy.

### Cell culture

ERα, ERβ and GPER-expressing MCF-7 breast cancer cell lines were used to investigate the effect of ICI 182,780 and of G-1 on cells that express these three receptors. The SKBr3 cell line, which expresses GPER but not ERα, was used to investigate whether ICI and G-1’s effect on the formation of acidic compartments was present only in cells expressing ERα. ^5^ These cells were kept at 37 ^o^ C and 5% carbon dioxide in serum-free phenol red DMEM/F12, supplemented with 10% FBS, 100U/mL penicillin and 100µg/mL streptomycin. They were also plated into serum free phenol red DMEM/F-12 for 24 hours.

### Treatments to verify cell viability and autophagy

The concentrations used in the experiments were based on the literature. Estrogen receptor can respond to concentrations in the picomolar and nanomolar ranges. Thus, for the components that act on these receptors, we used nanomolar concentrations to shorten the treatment duration required for effects to be observed, since induction of autophagy usually occurs before any reduction in viability can be detected. The antiproliferative effects of ICI 182,780 can be observed at concentrations as low as 1nM and are treatment-duration- and concentration-dependent. ^4 , 10^ Studies show that G-1 at 100nM leads to GPER activation through rapid pathways and signaling pathways that trigger gene transcription, although lower concentrations have been reported. ^11^ In these experiments, cells were also treated with RAP as positive control for autophagy induction, due to its mTOR inhibition. Concentrations in the literature range between 20nM and 10µM, for 24-hour treatments in breast cancer cell lines. ^12 , 13^ For this study, we used 1µM RAP, which has been shown to induce LC3-II formation on the membrane of autophagosomes.

Since ICI is an ER antagonist, as counterproof to rule out the activation of ERs, we used E2, an ER and GPER agonist, and PPT, a selective ERα agonist. The E2 concentrations to activate ERs are in the picomolar and nanomolar ranges, such as 0.1nM to 10nM. ^4^ In the literature, PPT concentrations range from 5 to 200nM and, therefore, we chose to use intermediate concentrations, such as 10nM and 100nM. ^14^

### Western blot for assessment of estrogen-receptor alpha and G-protein-coupled receptor (GPER) expression

Cells were plated into six-well plates and kept in medium until the day of the experiment. We performed the total protein extraction protocol with lysis buffer containing 10mM Tris, pH 7.4, 10mM NaCl, 3mM MgCl _2_ , 0.5% Nonidet P40, protease inhibitor cocktail, 1mM PMSF, 2mM Na _3_ VO _4_ , 50mM NaF, 10mM Na _2_ P _2_ O _7_ (Sigma Chemical Co.). Next, extracts were centrifuged (13.200rpm for 5 minutes at 4°C), and the supernatant was collected. Approximately 50μg of proteins were separated by polyacrylamide gel electrophoresis and transferred onto a polyvinylidene fluoride (PVDF) membrane. Blocking of nonspecific sites was performed by incubation with non-fat skim milk (5%) in TBS-Tween (1%) buffer for 1 hour, at room temperature. Membranes were incubated overnight at 4°C with primary antibodies (diluted with 2% skim milk in TBS-Tween buffer) for ERα (sc 8002, Santa Cruz Biotechnology) and GPER (ab39742, Abcam). A specific, horseradish peroxidase (HRP) labelled antibody was used. The product of the reaction was developed with ECL (PerkinElmer), and images were captured with a UVITEC digital photo documenter (Cambridge). Next, membranes were subjected to a stripping protocol and incubated with anti-alpha tubulin (1:10,000, Sigma-Aldrich Biotechnology), for internal control labelling.

### Cell viability assessment using the MTT assay

The MTT assay is based on the activity of mitochondrial enzymes from viable cells, leading to conversion of yellow MTT tetrazolium salt into a dark blue formazan product. ^15^ The MCF-7 were plated in triplicate in 96-well plates, at a density of 10 ^4^ cells/well. After 24 hours, the FBS was removed and cells were kept in DMEM/F12 for 24 hours, then treated with ICI, G-1 and RAP for 24 hours, 48 hours and 72 hours, and compared with a no-treatment control (CTR) group kept only in DMEM/F12. For reading, we added 50µg/mL MTT (diluted with phosphate-buffered saline − PBS) to each well, and proceeded with incubation at 37°C, for 3 hours. The medium was removed and 100% alcohol was added to each well. The reading was performed on a plate spectrophotometer at a wavelength of 595nm.

### Transfection of MCF-7 cells for GFP-LC3 expression

During elongation of the autophagic vesicle, LC3 (light chain 3) is recruited. The LC3 protein, homogeneously spread throughout the cytosol as LC3-I, is specifically recruited to the membrane of autophagosome vesicles, changing into LC3-II. When LC3 fuses with the green fluorescent protein (GFP), induction of autophagy can be observed as LC3 accumulates in autophagosomes, and green puncta GFP can be seen on fluorescence microscopy. ^16^

Cells were plated into 35mm plates with a 25mm cover slip treated with polyornithine (0.01mg/mL). After adhesion, cells were transfected with 0.5µg pCLPCX-GFP-LC3 plasmid, which contains the gene encoding for the LC3 protein, conjugated with the gene encoding for GFP. For transfection, we used 0.5µg of the plasmid mixed with 3μL FuGENE ^®^ HD (Fugent, LLC., USA) in 100μL DMEM/F12. Cells were incubated with the reagent mix in 1.5mL DMEM/F12 supplemented with 10% FBS, 100U/mL penicillin and 100µg/mL streptomycin, kept at 37°C and 5% carbon dioxide. On the following day, cells were carefully washed with FBS and incubated with non-supplemented DMEM/F12 for another 24 hours, adding up to a total of 48 hours for protein expression.

### Fluorescence microscopy to assess autophagy resulting from GFP-LC3 expression

Transfected cells were analyzed on a Nikon TE300 (Nikon Osaka, Japan) fluorescence microscope equipped with a CoolSNAP™ high-resolution digital camera (Roper Sci, Princeton Instruments, USA), and a cooling system to reduce noise and improve image resolution. Images were obtained every 30 minutes, with a maximum duration of 4 hours for each experiment. Forty-times magnification was used to perform the experiments. Excitation took place at a wavelength of 488nm and emission at 505nm.

Since autophagy is a basal process, the increase in GFP-LC3 puncta is due to the presence of an autophagy-inducing stimulus. To quantify the puncta, we established a cutoff point derived from the control sample (non-treated cells, kept in DMEM/F12). For each control cell, we calculated the difference between the baseline and peak number of puncta, irrespective of time, with a maximum observation duration of 4 hours.

### Detection of acidic compartments by flow cytometry

Cells were stained with AO for estimation of the number of acidic vesicular organelles by flow cytometry, as a marker of the final steps of the autophagy process, ^17^ since AO, in its protonated form, accumulates inside acidic vesicles.

The cells were plated in six-well plates and kept in FBS-free DMEM/F12. In the experiments, cells were treated with ICI, G-1, RAP, E2 and PPT, for 24 or 48 hours, compared with the control (CTR) group. Next, they were trypsinizated with addition of 1mL PBS, transferred into tubes, and centrifuged at 1,300rpm for 8 minutes. The supernatant was discarded, and cells were resuspended in 400µL AO diluted with PBS (100ng/mL). Cells were incubated for 15 minutes at room temperature and protected from light. Measures were obtained by flow cytometry (FACSCalibur, BD, Biosciences). The wavelength for excitation was 488 nm, fluorescence was detected at 510 to 530nm (green fluorescence, FL1) and 650nm (red fluorescence, FL3). The data were generated by the CellQuest™ software program and analysed on WinMDI 2.9.

### Statistical analysis

The results obtained were subjected to statistical analysis by one-way analysis of variance (ANOVA), followed by Turkey or Bonferroni’s post-hoc test, and Student’s *t* test. The data were expressed in histograms as mean±standard error of the mean. Analyses were conducted in the GraphPad Prism software program, version 4.0.

## RESULTS

### Expression of estrogen receptors in MCF-7 and SKBr3 cell lines

**Figure 1:**
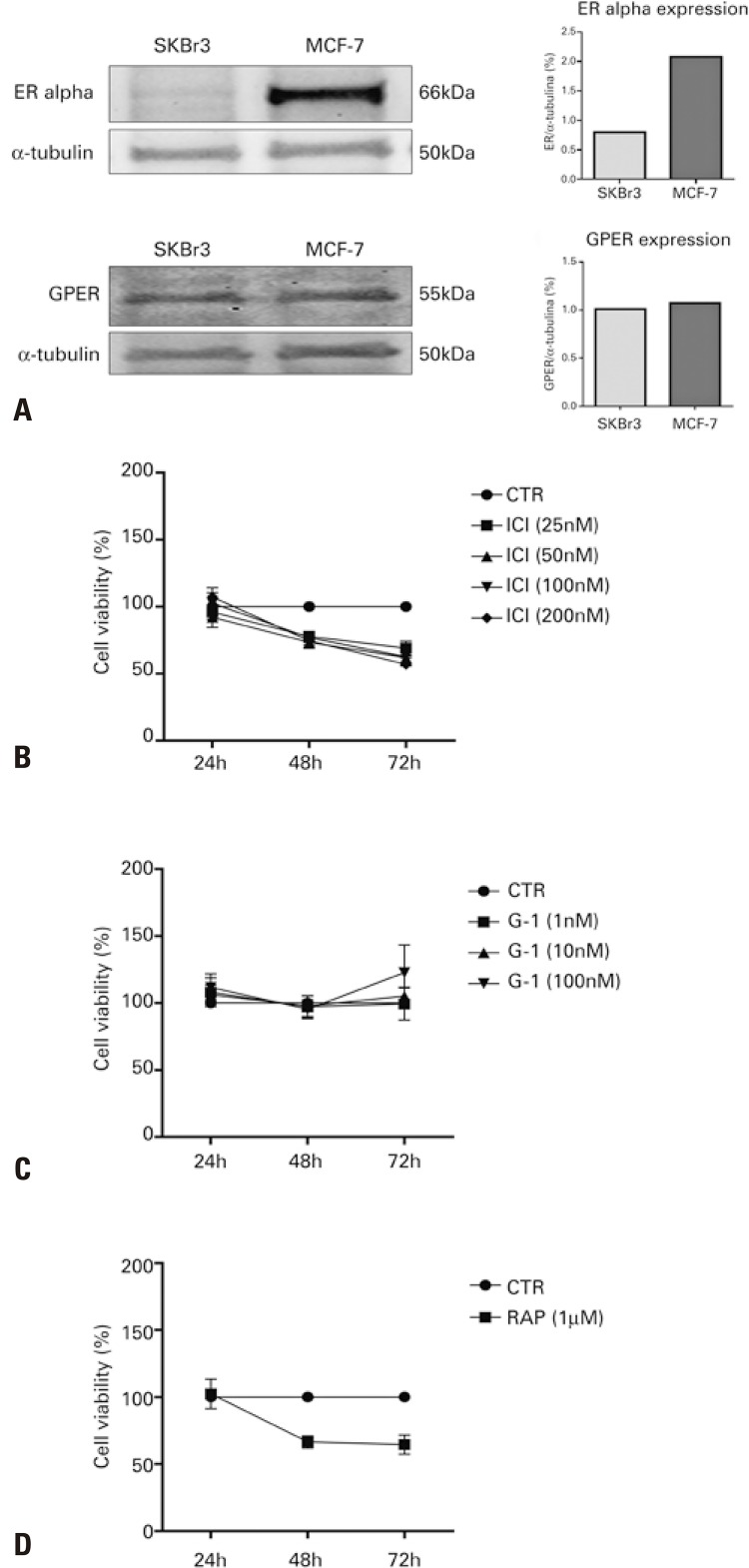
Expression of estrogen receptors in MCF-7 and SKBr3 cell lines and cell viability by MTT in MCF-7 cells. In A, expression of estrogen receptors alpha and G-protein-coupled receptors (GPER) in MCF-7 and SKBr3 cells. Representative autoradiograms show that estrogen receptor alpha is differentially expressed in the two cell lines, whereas the G-protein-coupled receptor (GPER) is expressed at similar levels. In B, C and D, cell viability by MTT in MCF-7 cells treated with ICI (B), G-1 (C) and rapamycin (D) for 24, 48 and 72 hours in FBS-free DMEM/F12. The data represent the mean±standard error of the mean of four independent experiments (24 hours and 48 hours) and three experiments in triplicate (72 hours). One-way analysis of variance (ICI and G-1) with multiple comparisons by Tukey’s test. Unpaired *t* test (rapamycin *versus* CTR). P<0.05 represents a significant difference *versus* the Control Group (no treatment). ICI and rapamycin reduced cell viability in MCF-7 after 48 and 72 hours. G-1 did not affect cell viability after 24, 48 or 72 hours ERα: estrogen receptor alpha; CTR: Control Group (no treatment); RAP: rapamycin; ICI: fulvestrant.

Initially we conducted a Western blot assay to identify any receptors differentially expressed in the two different breast cancer cell lines. As shown in figure 1A , ERα was highly expressed on MCF-7 cells, but not significantly on SKBr3 cells. The GPER receptor, in turn, was expressed in similar quantities in both cell lines.

### Cell viability assay of MCF-7 and SKBr3 cells

**Figure 2:**
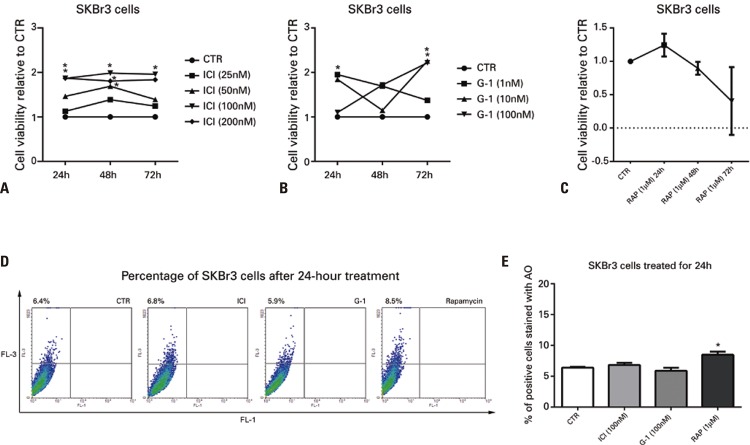
Effects of ICI, G-1 and rapamycin in SKBr3 cells. Cell viability by MTT in SKBr3 cells treated with ICI (A), G-1 (B) and rapamycin (C) for 24, 48 and 72 hours in FBS-free DMEM/F12. The data represent the mean of an experiment in triplicate. p<0.05* represents a statistical difference from control, considering the standard deviation of the sample. None of the compounds (ICI, G-1 and RAP) was capable of reducing cell viability in SKBr3 cells. In D and E, staining of SKBr3 cells with acridine orange after treatment with ICI (100nM), G-1 (100nM) and rapamycin (1µM) for 24 hours shows that ICI and G-1 did not induce formation of acidic compartments in SKBr3, a cell line that does not express estrogen receptor alpha. In D, fluorescence detection charts for green and red fluorescence. In E, histograms show mean values (±standard error of the mean) in percentage of cells stained with acridine orange. One-way analysis of variance, followed by Bonferroni’s test (n=4). Control Group (no treatment). In E, *p<0.05 represents a statistical difference of rapamycin *versus* Control Group (no treatment) and G-1 CTR: Control Group (no treatment); RAP: rapamycin; ICI: fulvestrant; AO: acridine orange.

A viability assay was performed to test the effect of ICI, G-1 and RAP (as an autophagy inducer by mTOR inhibition) on the growth of MCF-7 ( Figure 1B to 1D ) and SKBr3 ( Figure 2A to 2C ) cells. The decrease in the number of cells could be due to inhibition of proliferation, or cell death. ^15^ To proceed with the autophagy assessment, we first checked how long it took for cell viability to decrease after treatment with ICI, G-1 and RAP in MCF-7 cells. Twenty-four-hour treatments with ICI (25, 50, 100 and 200nM) produced no changes in cell viability. However, when used for 48 and 72 hours, ICI led to decreased viability (p<0.05). When comparing the response to ICI at different concentrations, there was no difference in reduction of cell viability, however at 100nM, the variability between samples was smaller ( Figure 1B ). There was no difference between the 48- and the 72-hour treatment (p>0.05). In treatments with GPER agonist G-1 at 1nM, 10nM and 100nM, for 24, 48 and 72 hours ( Figure 1C ), there was no decrease in the number of cells (p>0.05). To use RAP as positive control in autophagy assays, we tested its effect on viability to determine the best treatment duration preceding observable decreased cell viability. In the 24-hour treatment with RAP (1µM), no changes were observed (p>0.05); however, treatments for 48 and 72 hours (p<0.05 *versus* control) led to decreased viability ( Figure 1D ). There was no difference between the 48- and 72-hour treatment durations (p>0.05).

When assessing cell viability of SKBr3 cells ( Figure 2A ), differently from MCF-7 cells, ICI showed a time- and concentration-dependent increase in viability compared with the Control Group. We observed increased cell viability at 50nM (48 hours), 100nM (24, 48 and 72 hours) and 200nM (24 and 48 hours). Treatment with G-1 in SKBr3 cells also increased cell viability at certain concentrations and treatment durations (at 1nM for 24 hours, and at 10nM and 100nM for 72 hours; Figure 2B ). RAP did not affect cell viability in SKBr3 cells, irrespective of duration ( Figure 2C ). To investigate the absence of autophagy, SKBr3 cells, which do not express ERα, were treated with ICI (100nM), G-1 (100nM) and RAP (1μM) for 24 hours ( Figures 2D and 2E ). Cells were stained with AO and analyzed by flow cytometry. The data showed that ICI and G-1 did not increase the number of acidic vesicular organelles when compared with CTR (p>0.05) and RAP (p<0.05), suggesting that, since there was no decrease in viability, the absence of autophagy could explain this result.

### Effect of treatment with ICI 182,780 and G-1 on autophagy induction based on GFP-LC3 puncta and formation of acidic vesicular organelles in MCF-7 cells

During formation of autophagosomes, LC3 is specifically translocated onto the autophagosome membrane. ^16^

**Figure 3:**
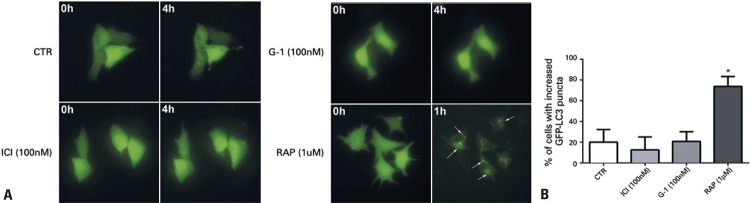
Autophagy induced by rapamycin based on GFP-LC3 puncta in MCF-7 cells. ICI and G-1 did not induce autophagy within the time observed (4 hours). In A, images obtained by fluorescence microscopy of GFP-LC3-expressing MCF-7 cells stimulated with rapamycin (1µM), ICI (100nM) and G-1 (100nM). Cells were kept in DMEM/F12 (CTR) and visualized every 30 minutes, over the course of 4 hours (40X magnification). In B, the histogram represents the quantification of cells with increased GFP-LC3 puncta, within 4 hours in MCF-7 cells. The mean±standard error of the mean was calculated from five independent control experiments. The upper limit was obtained (mean + two times the standard error of the mean) and established as cutoff which, in this case, was equal to an increase of nine puncta per cell. For each experiment, we calculated the percentage of cells with an increase greater than this limit. One-way analysis of variance, followed by Bonferroni’s test. Mean values obtained from at least three independent experiments. The ICI, G-1 and rapamycin groups were compared with the Control Group * p<0.05 represents a significant difference. CTR: Control Group (no treatment); RAP: rapamycin; ICI: fulvestrant.

GFP-LC3-expressing MCF-7 cells were visualized for 4 hours after treatment with ICI (100nM) and G-1 (100nM) ( Figure 3 ). Rapamycin (1μM) was used as positive control. Results show that treatment with ICI and G-1 did not induce GFP-LC3 translocation indicating increased autophagy when compared with CTR. Rapamycin contrarily, induced formation of autophagosomes, shown by GFP-LC3 translocation and increased puncta ( Figure 3A ).

**Figure 4:**
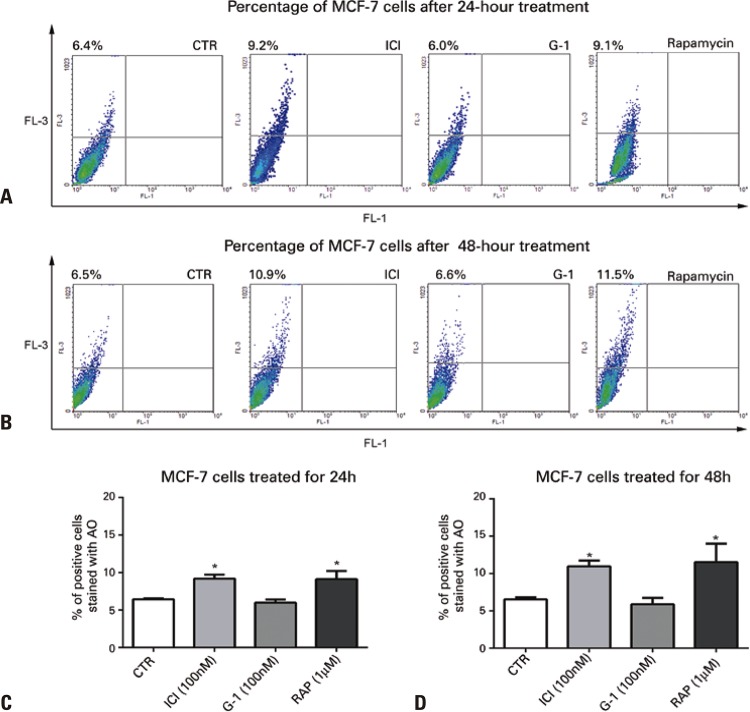
Effect of ICI, G-1 and rapamycin on formation of acidic vesicular organelles in MCF-7 cells. ICI and rapamycin induce formation of acidic compartments within 24 and 48 hours in MCF-7 cells, whereas G-1 did not affect this process. Histograms show the mean value±standard error of mean, in percentage of cells stained with acridine orange and analyzed by flow cytometry. MCF-7 cells were treated with ICI (100nM), G-1 (100nM) and rapamycin (1µM) for 24 hours (A and C) and 48 hours (B and D). One-way analysis of variance, followed by Bonferroni’s test. In A, n=6; in B, n=5 *p<0.05 represents a significant difference of ICI *versus* (Control Group, no treatment, and G-1) rapamycin (control group, no treatment, and G-1). CTR: Control Group (no treatment); RAP: rapamycin; ICI: fulvestrant; AO: acridine orange.

As previously described, ^9^ seeing that autophagy is a process that can take place as a survival mechanism in the presence of a stressor, we investigated whether these effects took place before any potential reduction in viability. Since results showed there was no decrease in proliferation within 24 hours, but rather within 48 hours, we used these treatment durations to investigate the effects of ICI and G-1 on formation of acidic compartments by AO staining and detection by flow cytometry ( Figure 4 ). Results showed that treatment with ICI (100nM) increased the number of acidic compartments, for both 24 and 48 hours of treatment, similarly to RAP (1μM). Both treatments were significantly different (p<0.05) from CTR and there was no difference between ICI and RAP (p>0.05), nor between 24 and 48 hours of treatment ( Figure 4 ). On the other hand, no changes were observed in the number of acidic compartments (p>0.05) in cells treated with G-1 ( Figure 4 ).

### Effect of 17β-estradiol and PPT on formation of acidic compartments in MCF-7 cells as an indicator of autophagy

**Figure 5:**
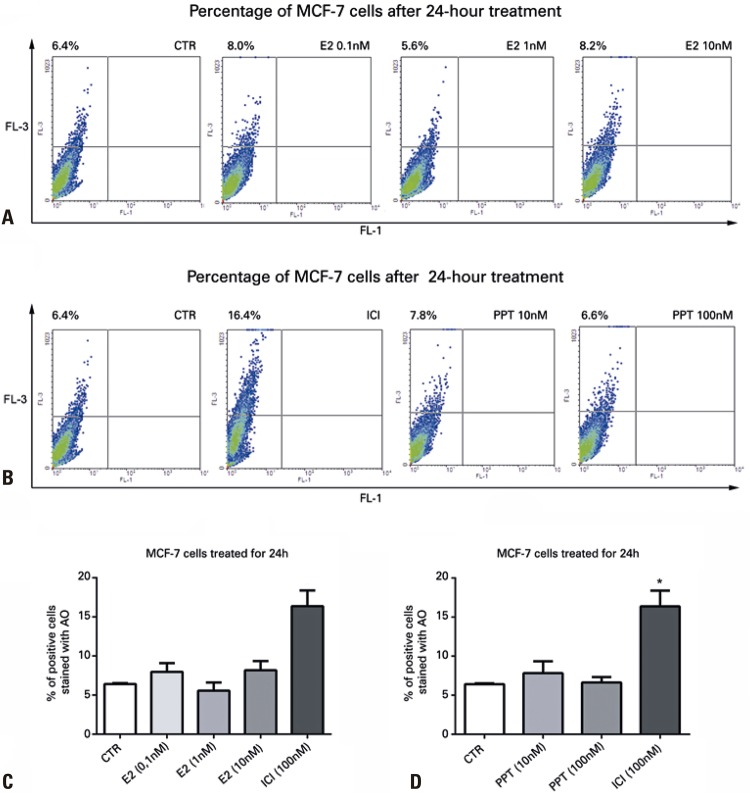
Detection of acidic vesicular organelles by flow cytometry in MCF-7 cells stained with acridine orange after treatment with compounds for 24 hours. The activation of estrogen receptors with 17β-estradiol, as well as the selective activation of estrogen receptor alpha with PPT, does not induce acidic compartment formation in MCF-7 cells. In A and C, MCF-7 cells treated with 17β-estradiol at 0.1nM, 1nM and 10nM. In B and D, MCF-7 cells treated with PPT at 10nM and 100nM. In MCF-7 cells, the effects were compared with those of ICI (100nM). In A and B, fluorescence detection charts for green and red fluorescence. In C and D, histograms show mean values±standard error of the mean, in percentage of cells stained with acridine orange. One-way analysis of variance, followed by Bonferroni’s test (n=4). In C and D, *p<0.05 means a statistical difference in comparison with the other groups * p<0.05 means a statistical difference in comparison with the other groups. CTR: Control Group (no treatment); E2: 17β-estradiol; ICI: fulvestrant; AO: acridine orange.

Since the decrease in cell viability and the increase in acidic compartments in MCF-7 cells occurred after treatment with ICI but not with G-1, these effects could be associated with ER antagonism, without involving GPER activation. To investigate how ER antagonists affect autophagy, MCF-7 cells were treated for 24 hours with E2 (0.1nM, 1nM and 10nM) and compared with ICI (100nM). Results show that the different E2 concentrations were not capable of increasing the number of acidic compartments in cells (p>0.05 compared with CTR; Figures 5A and 5C ). Since E2 acts differently on different ERs, MCF-7 cells were treated for 24 hours with PPT (10nM and 100nM), a specific ERα agonist that also failed to increase the number of acidic compartments (p>0.05 compared with control) ( Figures 5B and 5D ).

## DISCUSSION

Autophagy is a cell mechanism that can potentially eliminate intracellular components following an insult or build-up of defective proteins or organelles. ^9^ Abnormal autophagy may occur in cancer cells and after chemotherapy. In breast cancer, evidence shows that proteins involved in autophagy, such as beclin-1, regulate cell growth and interfere in estrogen signaling. ^18^

Studies with ER antagonists, such as ICI and tamoxifen, in breast cancer cells show increased autophagy ^10 , 18 - 22^ which can lead to breast cancer resistance and proliferation. ICI is knowingly an ERα and ERβ antagonist, however, there are reports that in models in which ICI has no effect or agonist effect, its action supposedly results from GPER activation ^5^ Therefore, in this study, we initially compared the cytotoxic/antiproliferative actions of ICI with G-1, a selective GPER agonist. We verified a potential antiproliferative action of ICI in ER-expressing MCF-7 cells, with 48- and 72-hour treatments, considering that there was no death of these cells under these experimental conditions in parallel trials performed (data not shown). We know that ICI leads to decreased ERα activity ^23^ and its antiproliferative effect is associated with blocking of classic ERs, leading ER-positive cells to cell death by apoptosis. ^24^

As for the role of GPER in cell proliferation, despite our results showing that selective GPER activation by G-1 did not affect the parameters assessed, previous studies have shown the antiproliferative and apoptotic effects of G-1 on MCF-7, at concentrations equal to or higher than 1µM for at least 72 hours of treatment. ^25 , 26^ On the other hand, it has been shown that the GPER pathway may be involved in the aggressive behavior of breast tumors by regulation of expression of the aromatase enzyme, after GPER inhibition in tamoxifen-resistant MCF-7 cells. ^27^ Thus, GPER could be a potential target for breast cancer management, and its role in breast tumor growth and tumor resistance after antiestrogen therapy must be further investigated.

To investigate the role of ER and GPER in autophagy, we studied the effect of ICI and G-1 on GFP-LC3 translocation, which corresponds to an estimate of the autophagy process. Despite ICI not leading to GFP-LC3 translocation in this study, this may be due to the short exposure time of 4 hours, whereas more time would be required to induce autophagy. ^10 , 28^ Also, the autophagic pathways induced may not involve LC3 translocation. ^7^ Selective GPER activation with G-1, in turn, did not lead to autophagy by LC3 translocation. The effects of G-1 on autophagy are still unclear and under the experimental conditions used herein, no significant changes were observed. As for the role of GPER in autophagy, it was reported that G-15, a GPER antagonist, can induce ER-independent autophagy in human head and neck cancer cell lines, irrespective of GPER expression levels. ^29^ The role of GPER in cancer proliferation has been greatly explored, however no inter-relation between this pathway and autophagy, an important phenomenon related with cell survival, has been identified.

Experiments with ICI in MCF-7 cells have shown increased staining with AO, indicative of an increased number of acidic compartments. This effect was not seen in the SKBr3 cell line, which does not express ERα. Studies have shown ^10 , 28^ that ICI can induce autophagy in MCF-7 cells by other methods, corroborating our findings. In addition to investigating LC3 translocation, these studies also addressed the decrease in p62, another indicator of induced autophagy. This protein is required for formation of ubiquitinated protein aggregates, targeted at and incorporated into autophagosomes and, ultimately, degraded in autolysosomes. ^16^ ICI is known to reduce ERα activity by inducing degradation of this receptor by the ubiquitin-proteasome system. ^23^ The relation between the ubiquitin-proteasome system and autophagy in molecule uptake and degradation processes must be further investigated. ^30^ Also, evidence points to an interaction between ERα and beclin 1, one of the primary proteins involved in formation of autophagosomes, ^18^ which indicates that ICI-induced autophagy is mainly related with ICI’s effect as an ERα antagonist.

MTT results show that the viability of SKBr3 cells was not affected after treatment with RAP 1µM for 24, 48 and 72 hours. According to clinical trials, RAP appears to have a cytostatic effect, since the reduction in breast cancer proliferation is modest, however when combined with other chemotherapeutic agents, their synergistic effect is more effective. ^31^ Nevertheless, RAP induces formation of acidic vesicles, possibly by inducing autophagy, without, however, decreasing cell viability. Notwithstanding, in this study, RAP was used as positive control to assess the increase in acidic vesicular organelles, compared with ICI and G-1.

When comparing ICI and G-1 in SKBr3 cells, some of the treatments, depending on the respective duration and concentration, increased viability of these cells without any reduction in cell viability. GPER induction appeared to be a survival response in ER-negative cells. In parallel, there was no increased formation of acidic vesicles such as lysosomes, which correspond to the latest steps of macromolecular component degradation, and take place in the presence of cell stressors. Some studies ^11 , 32^ have reported pro-proliferative effects of G-1 and ICI in SKBr3 cells, showing that the absence of ERs, particularly ERα, and the presence of GPER can determine proliferative effects in estrogen-receptor-negative breast cancer cells, which explains why treatment with antiestrogens, such as tamoxifen and ICI, is not effective in this type of breast cancer.

Since the link between autophagy and cancer was established, many studies have attempted to understand the role of autophagy and how it relates with cancer and treatment response, investigating the connection between the autophagic pathway and cell proliferation and/or death. In breast cancer, studies identified that ER antagonists, such as ICI and tamoxifen, can promote autophagy in breast cancer cells. ^10 , 18 - 22^ Recent evidence shows that inhibiting autophagy can induce cell death, since autophagy is a survival mechanism, ^22 , 33 , 34^ and, in estrogen-receptor-positive breast cancer cells, ICI-induced autophagy seems to contribute to cancer resistance. ^10^ Since ICI also acts by activating GPER, the possibility of the GPER pathway being involved in survival by autophagy must also be investigated. Thus, it is important to assess the role of this receptor in the survival mechanisms of breast cancer, since it could potentially become a relevant target for cancer treatment.

## CONCLUSION

A relevant issue in breast cancer therapy is the resistance developed after therapy with antiestrogens such as ICI. One of its targets, the G-protein-coupled estrogen receptor, supposedly has a major role in the proliferation/viability of cancer cells, and its effect seems to vary based on surrounding conditions. The activation of autophagy is currently widely investigated as a cell protection mechanism. The effect of ICI in increasing acidic compartments in ER-positive cells seems to be greatly associated with its ability to inhibit estrogen receptors, whereas the G-protein-coupled estrogen receptor does not seem to be involved. These data may be important to explain cell death and resistance mechanisms, aiming to develop new treatment alternatives.
