# Supplementary material for: Effects of ICI 182,780, an ERα and ERβ antagonist, and G-1, a GPER agonist, on autophagy in breast cancer cells
Source: Einstein (Sao Paulo). 2020 Apr 13;18:eAO4560. doi: 10.31744/einstein_journal/2020AO4560 (PMC7155941; doi:10.31744/einstein_journal/2020AO4560)
Supplement: Translation

# Translation: Efeitos do antagonista seletivo do REα e do REβ ICI 182,780 e do agonista de GPER G-1 no processo de autofagia em células de câncer de mama

**Authors:** Mari Luminosa Muler, Fernanda Antunes, Gabriel Cicolin Guarache, Rafaela Brito Oliveira, Rodrigo Portes Ureshino, Claudia Bincoletto, Gustavo José da Silva Pereira, Soraya Soubhi Smaili

**Affiliations:** 1 Brasil Departamento de Farmacologia, Escola Paulista de Medicina, Universidade Federal de São Paulo, São Paulo, SP, Brasil .; 2 Brasil Departamento de Ciências Biológicas, Universidade Federal de São Paulo, Diadema, SP, Brasil .

**Keywords:** Autofagia, Neoplasias da mama, Estrógenos, Receptores estrogênicos, Receptores acoplados a proteínas-G, Fulvestranto, Proliferação celular, Células MCF-7

## Abstract

**Objetivo:**

Avaliar o efeito dos compostos ICI 182,780 (fulvestranto), um antagonista seletivo dos receptores de estrógeno alfa/beta (REα/REβ), e do G-1, um agonista seletivo de receptores de estrógeno acoplados a proteínas-G (GPER), na possível indução de autofagia em linhagens de câncer de mama MCF-7 e SKBr3, bem como o efeito de G-1 na viabilidade celular.

**Métodos:**

A viabilidade celular de células MCF-7 e SKBr3 foi avaliada pelo ensaio com MTT. Para investigar a indução da autofagia, células MCF-7 foram transfectadas com GFP-LC3, um marcador de autofagossomos, e analisadas por microscopia de fluorescência em tempo real. As células MCF-7 e SKBr3 foram incubadas com o indicador de compartimentos ácidos laranja de acridina e analisadas por citometria de fluxo como indicativo para autofagia.

**Resultados:**

Em células MCF-7, o ICI 182,780 e rapamicina após 48 horas levaram à diminuição da viabilidade celular, enquanto o G-1 não alterou a viabilidade no mesmo período de tratamento. Nem o ICI 182,780 e nem o G-1 induziram aumento na pontuação de GFP-LC3 em células MCF-7 até 4 horas. Já os ensaios de citometria de fluxo demonstraram que ICI 182,780 levou ao aumento de compartimentos ácidos em células MCF-7. O G-1 não aumentou estes parâmetros em ambas as linhagens. Por outro lado, ICI 182,780 e G-1 não induziram à redução da viabilidade em células SKBr3 e nem à formação de compartimentos ácidos, como etapa final do processo autofágico.

**Conclusão:**

O aumento de compartimentos ácidos pelo ICI 182,780 em células de câncer de mama positivas para receptores de estrógeno parece estar associado com seu efeito inibidor de receptores de estrógeno, mas sem o envolvimento de GPER. A compreensão desses mecanismos pode direcionar estudos sobre o envolvimento dos receptores nos processos celulares de resistência do câncer de mama.

## INTRODUÇÃO

O câncer de mama é uma das principais causas de morte em mulheres, tanto no Brasil quanto em outros países.^1^ Em alguns casos, o tratamento pode levar à cura, e um dos protocolos mais adotados envolve a excisão cirúrgica, seguida de quimioterapia/radioterapia. Nos casos de câncer de mama responsivos a hormônios,^2^ a terapia endócrina consiste em inibir a síntese do estrógeno ou interferir na sinalização mediada por este esteroide.^3^ Nestes casos, para o tratamento padrão, é utilizado o tamoxifeno, um modulador seletivo de receptores de estrógeno (RE), além do tratamento com ICI 182,780 (fulvestranto − referido neste artigo como ICI) e os inibidores de aromatase,^4^ para diminuir a estimulação estrogênica, que pode promover a síntese proteica e a proliferação celular.

Os RE primeiramente descritos foram o alfa (REα) e o beta (REβ), cujo principal mecanismo é sua ação como fator de transcrição. Posteriormente, foi descrito um RE com sete domínios transmembranosos acoplados à proteína G (GPER), cujo padrão de expressão pode indicar a agressividade do tumor quando associada à expressão de RE.^5^ Apesar da ação antagonista de ICI ser sobre os receptores clássicos (REα e REβ), sua ação sobre o GPER é agonista,^5^ e as vias de sinalização ainda precisam ser elucidadas. Evidências têm indicado que a expressão de REα pode modular a resposta de células de câncer frente a diferentes tratamentos, por meio da autofagia.^6 , 7^ A macroautofagia, referida como “autofagia”, é uma via lisossomal de reciclagem de macromoléculas e organelas celulares, que são sequestradas em autofagossomos, circundados por membrana dupla. Estas se fundem com lisossomos, formando os autolisossomos, nos quais ocorre a degradação.^8^

Estudos demonstraram que a ativação de autofagia em células normais pode evitar a formação do tumor, e a inibição de autofagia pode ser benéfica em tumores estabelecidos. Por outro lado, drogas citotóxicas utilizadas no tratamento de câncer mostraram ser indutoras de autofagia e podem desencadear morte celular em células deficientes em apoptose.^9^ Pelo fato da autofagia ter papel duplo na tumorigênese e na progressão tumoral, a compreensão desses mecanismos pode trazer entendimento de como atuar na descoberta de possíveis alvos de tratamento no câncer de mama.

## OBJETIVO

Avaliar o efeito dos compostos ICI 182,780 (fulvestranto), um antagonista seletivo dos receptores de estrógeno alfa/beta (REα/REβ), e do G-1, um agonista seletivo de receptores de estrógeno acoplados a proteínas-G (GPER), na possível indução de autofagia em linhagens de câncer de mama MCF-7 e SKBr3, bem como o efeito de G-1 na viabilidade celular.

## MÉTODOS

Este trabalho foi aprovado pelo Comitê de Ética da Universidade Federal de São Paulo, com número do parecer 1748/10.

### Reagentes

DMEM/F12, soro fetal bovino (SFB), penicilina/ estreptomicina e tripsina/ácido etilenodiamino tetra-acético (EDTA) 0,5% foram obtidos da Invitrogen™ do Brasil (St. Louis, MO, USA). ICI 182,780 (AstraZeneca do Brasil; Cotia, São Paulo, Brasil), 1-[4-(6-bromobenzo[1,3]dioxol-5-il)-3a,4,5,9b-tetrahidro-3Hciclopenta[c]quinolin-8-il]-etanona (G-1; Calbiochem^®^; Merck Biosciences, Darmstadt, Alemanha), 3-benzoato 17β-estradiol (17β-estradiol, E2) (Sigma Chemical Co.; St Louis, MO, USA) e 4,4’,4’’-[4-propil-(1H)-pirazol-1,3,5-triil]trisfenol (PPT). Rapamicina (RAP) e brometo de [3-(4,5-dimetiltiazol-2il)-2,5-difenil tetrazólio (MTT) foram obtidos da Sigma Aldrich (St. Louis, MO, USA). Laranja de acridina (LA) foi obtida da Molecular Probes (Eugene, OR, USA). O plasmídeo GFP-LC3 foi proveniente do *National Institute for Infectious Diseases* , nos Estados Unidos, e *Istituto di Ricovero e Cura a Carattere Scientifico L. Spallanzani* , Roma, Itália.

### Cultura de células

Foram utilizadas linhagens de células de câncer de mama MCF-7, que expressam REα, REβ e GPER, para verificar qual o efeito de ICI 182,780 e G-1 nas células que possuem os três tipos de receptores. A linhagem SKBr3, que expressa GPER mas não expressa REα, foi utilizada para verificar se os efeitos de ICI e G-1 na formação de compartimentos ácidos ocorriam apenas nas células que possuem REα.^5^ As células foram mantidas em 37^°^C e 5% dióxido de carbono em meio DMEM/F12 livre de vermelho de fenol e suplementado com 10% SFB, 100U/mL de penicilina e 100µg/mL de estreptomicina. Foram também plaqueadas em meio DMEM/F-12 livre de vermelho de fenol por 24 horas.

### Tratamentos na verificação de viabilidade celular e autofagia

As concentrações utilizadas nos experimentos foram baseadas na literatura. Os RE podem responder a concentrações na ordem de picomolar e nanomolar. Assim, para os componentes que possuem ação nesses receptores, foram utilizadas concentrações na ordem de nanomolar, para que os efeitos fossem observados em tempos menores de tratamento, já que o processo de indução de autofagia ocorre normalmente antes de se detectar a redução na viabilidade. Os efeitos antiproliferativos de ICI 182,780 podem ser observados a partir de concentrações como 1nM e são dependentes do tempo de tratamento, assim como da concentração.^4 , 10^ Estudos demonstram que G-1 na concentração de 100nM leva à ativação de GPER por vias rápidas e vias de sinalização que levam à transcrição de genes, embora concentrações menores também tenham sido relatadas.^11^ Nos experimentos, as células também foram tratadas com RAP como controle positivo na indução de autofagia, por sua inibição em mTOR. As concentrações utilizadas na literatura variam de 20nM até 10µM, em tratamentos por 24 horas, em linhagens de células de câncer de mama.^12 , 13^ Para este estudo, utilizou-se a concentração de 1µM de RAP descrita como capaz de levar à formação de LC3-II na membrana de autofagossomos.

Como o ICI é um antagonista de REs, como contraprova para descartar a ativação de REs, utilizou-se E2, que é agonista de REs e GPER, e PPT, um agonista seletivo de REα. As concentrações de E2 para ativar REs variam na ordem de picomolar e nanomolar, como 0,1nM até 10nM.^4^ Na literatura, são utilizadas concentrações de PPT desde 5 a 200nM, então, optou-se por utilizar concentrações intermediárias, como 10nM e 100nM.^14^

### *Western blot* para avaliar a expressão dos receptores de estrógeno alfa e receptores acoplados a proteínas-G

As células foram plaqueadas em placas de seis poços e mantidas em meio de cultura até o dia do experimento. Foi realizado o protocolo de extração de proteínas totais com tampão de lise contendo 10mM Tris, pH 7,4, 10mM NaCl, 3mM MgCl_2_, 0,5% Nonidet P40, coquetel de inibidores de proteases, 1mM PMSF, 2mM Na_3_VO_4_, 50mM NaF, 10mM Na_2_P_2_O_7_(Sigma Chemical Co.). Em seguida, os extratos foram centrifugados (13.200rpm, por 5 minutos, a 4^°^C), e o sobrenadante foi coletado. Aproximadamente 50μg de proteínas foram separados por eletroforese em gel de poliacrilamida e, posteriormente, transferidos para uma membrana de fluoreto de polivinilideno (PVDF). O bloqueio dos sítios inespecíficos foi realizado com incubação com leite desnatado (5%) em tampão TBS-Tween (1%) por 1 hora, à temperatura ambiente. As membranas foram incubadas durante a noite a 4°C com anticorpos primários (diluídos em leite 2% em tampão TBS-Tween) para o REα (sc 8002, Santa Cruz Biotechnology) e o GPER (ab39742, Abcam). Foi utilizado anticorpo secundário específico, marcado com peroxidase (HRP - *horseradish peroxidase* ). O produto da reação foi revelado com ECL (PerkinElmer), e as imagens foram capturadas em fotodocumentador digital UVITEC (Cambridge). Em seguida, as membranas foram submetidas a protocolo de *stripping* e incubadas com anti-alfa tubulina (1:10.000, Sigma-Aldrich Biotechnology), para a marcação do controle interno.

### Avaliação de viabilidade celular pelo ensaio de MTT

O ensaio de MTT se baseia na ação de enzimas mitocondriais de células viáveis que levam à conversão do sal tetrazólio MTT, amarelo, para um produto formazan azul escuro.^15^ Células MCF-7 foram plaqueadas em triplicatas em placas de 96 poços, com densidade de 10^4^ células/poço. Após 24 horas, o SFB foi retirado, e as células foram mantidas em DMEM/F12 por 24 horas, sendo posteriormente tratadas com ICI, G-1 e RAP por 24 horas, 48 horas e 72 horas e comparadas com um Grupo Controle (CTR), sem tratamento, mantido apenas em DMEM/F12. Para a leitura, foram adicionados 50µg/mL de MTT (diluído em tampão fosfato-salina − PBS) em cada poço e foi realizada incubação a 37^°^C, por 3 horas. O meio foi retirado e adicionou-se álcool 100% em cada poço. A leitura foi realizada em espectrofotômetro de placa, no comprimento de onda de 595nm.

### Transfecção de células MCF-7 para expressão de GFP-LC3

Durante a elongação da vesícula autofágica, ocorre recrutamento de LC3 ( *light chain* 3). A proteína LC3, encontrada dispersa homogeneamente no citosol na forma de LC3-I, é recrutada especificamente para a membrana de vesículas de autofagossomos, mudando para a forma LC3-II. A fusão da proteína LC3 com proteína fluorescente verde (GFP - *green fluorescent protein* ) permite observar a indução do processo de autofagia por meio do acúmulo de LC3 nos autofagossomo, sendo observada pontuação verde GFP em microscopia de fluorescência.^16^

As células foram plaqueadas em placas de 35mm contendo lamínula de 25mm tratada com poliornitina (0,01mg/mL). Após adesão, as células foram transfectadas com 0,5µg de plasmídeo pCLPCX-GFP-LC3, que contém o gene que codifica a proteína LC3 conjugada com o gene que, por sua vez, codifica a GFP. Para transfecção, foi utilizado 0,5µg do plasmídeo misturado com 3μL de FuGENE^®^HD (Fugent, LLC., USA) em 100μL de DMEM/F12. As células foram incubadas com a mistura dos reagentes em 1,5mL de meio DMEM/F12 suplementado com 10% de SFB, 100U/mL de penicilina e 100µg/mL de estreptomicina, e mantidas em 37^°^C e 5% de dióxido de carbono. No dia seguinte, as células foram cuidadosamente lavadas com PBS e incubadas com meio de cultura DMEM/F12 não suplementado por mais 24 horas, totalizando 48 horas para a expressão da proteína.

### Microscopia de fluorescência para avaliar autofagia decorrente de expressão de GFP-LC3

As células transfectadas foram visualizadas com microscópio de fluorescência Nikon TE300 (Nikon Osaka, Japão) acoplado a uma câmera digital CoolSNAP™ (Roper Sci, Princeton Instruments, USA) de alta resolução, com sistema de resfriamento para diminuição de ruídos e aumento da resolução. As imagens foram obtidas a cada 30 minutos, com tempo máximo de 4 horas para cada experimento. Os experimentos foram realizados com a objetiva de 40x. A excitação foi feita no comprimento de onda 488nm e emissão coletada em 505nm.

Como a autofagia é um processo basal, o aumento de pontuações da GFP-LC3 ocorre na presença de um estímulo indutor autofágico. Para fazer a quantificação dessas pontuações, foi estabelecido um limite de corte a partir do controle (células sem tratamento, mantidas em DMEM/F12). Para cada célula controle, foi calculada a diferença entre o número de pontuações no início e o número de pontuações máximo, independentemente do tempo, sendo tempo máximo de observação até 4 horas.

### Detecção de compartimentos ácidos por citometria de fluxo

As células foram marcadas com LA para estimar a quantidade de organelas vesiculares ácidas por citometria de fluxo, como indicativo das etapas finais do processo autofágico,^17^ uma vez que a LA, na forma protonada, acumula-se em vesículas ácidas.

Células plaqueadas em placas de seis poços foram mantidas em DMEM/F12 sem SFB. Nos experimentos, as células foram tratadas com ICI, G-1, RAP, E2 e PPT, por 24 ou 48 horas, e comparadas ao Grupo Controle (CTR). Depois foram tripsinizadas, adicionando-se 1mL de PBS, e transferidas para tubos, seguindo com centrifugação por 1.300rpm, por 8 minutos. O sobrenadante foi descartado, e as células foram ressuspensas em 400µL de LA diluída em PBS (100ng/mL). As células foram incubadas por 15 minutos em temperatura ambiente e protegidas da luz. As medidas foram obtidas por citometria de fluxo (FACSCalibur, BD, Biosciences). O comprimento de onda para excitação foi 488nm, e a detecção da fluorescência foi obtida por 510 a 530nm (fluorescência verde, FL1) e por 650nm (fluorescência vermelha, FL3). Os dados foram obtidos pelo programa CellQuest™ e analisados pelo programa WinMDI 2.9.

### Análise estatística

Os resultados obtidos foram submetidos à análise estatística por análise de variância (ANOVA) de uma via, seguida de pós-teste Tukey ou Bonferroni, e teste *t* de Student. Os dados foram expressos nos histogramas como média±erro médio padrão. As análises foram feitas no programa GraphPad Prism, versão 4.0.

## RESULTADOS

### Expressão de receptores de estrógeno nas linhagens MCF-7 e SKBr3

Inicialmente, foi realizado um ensaio de *Western blot* para verificar a presença dos receptores que estariam expressos diferencialmente nas duas linhagens de câncer de mama. Conforme demonstrado na figura 1A , o REα esteve expresso em grande quantidade em células MCF-7, mas não estava expresso de maneira significativa na célula SKBr3. Quanto ao receptor GPER, este encontrou-se expresso em quantidades similares em ambas as linhagens.

**Figura 1:** Expressão de receptores de estrógeno nas linhagens MCF-7 e SKBr3 e viabilidade celular por MTT em células MCF-7. Em A, expressão de receptores de estrógeno alfa e receptores acoplados a proteínas-G em células MCF-7 e SKBr3. Os autorradiogramas representativos mostram a expressão diferencial de receptores de estrógeno alfa nas duas linhagens, enquanto o receptor acoplado a proteínas-G se mantém em níveis similares. Em B, C e D, viabilidade celular por MTT de células MCF-7 tratadas com ICI (B), G-1 (C) e rapamicina (D) por 24 horas, 48 horas e 72 horas em meio DMEM/F12 sem soro fetal bovino. Os dados representam a média±erro médio padrão de quatro experimentos independentes (24 horas e 48 horas) e três experimentos (72 horas) feitos em triplicata. Análise de variância uma via (ICI e G-1) com múltiplas comparações por teste Tukey. Teste *t* não pareado (rapamicina *versus* CTR). Valor de p<0,05 foi considerado significativamente diferente ao Grupo Controle, sem tratamento. ICI e rapamicina diminuem a viabilidade celular em MCF-7 após 48 e 72 horas. G-1 não influenciou na viabilidade celular em 24, 48 ou 72 horas REα: receptor de estrógeno alfa; CTR: Grupo Controle (sem tratamento); RAP: rapamicina; ICI: fulvestranto.

### Ensaio de viabilidade celular das células MCF-7 e SKBr3

O ensaio de viabilidade foi realizado para testar o efeito do ICI, de G-1 e RAP (como indutor de autofagia, por inibição de mTOR) no crescimento de células MCF-7 ( Figura 1B a 1D ) e SKBr3 ( Figura 2A a 2C ). A diminuição no número de células pode ter sido devida à inibição da proliferação ou morte celular.^15^ Para prosseguir com a avaliação da autofagia, verificou-se, antes, em quanto tempo ocorreu a diminuição da viabilidade das células após os tratamentos com ICI, G-1 e RAP em células MCF-7 inicialmente. Nos tratamentos por 24 horas com ICI (25, 50, 100 e 200nM), não foram observadas alterações na viabilidade celular. Porém, nos tratamentos de 48 horas e 72 horas, o ICI levou à diminuição da viabilidade (p<0,05). Ao comparar a resposta entre as diferentes concentrações de ICI, não houve diferença na diminuição da viabilidade celular, mas na concentração de 100nM a variação entre as amostras foi menor ( Figura 1B ). Não houve diferença entre os tratamentos de 48 e 72 horas (p>0,05). Nos tratamentos com o agonista de GPER, G-1, nas concentrações de 1nM, 10nM e 100nM, por 24, 48 e 72 horas ( Figura 1C ), não foi observada diminuição no número de células (p>0,05). Para utilizar a RAP como controle positivo nos ensaios de autofagia, testou-se seu efeito na viabilidade, para determinar o melhor período de tratamento que precede à observação da diminuição de viabilidade celular. No tratamento de 24 horas com RAP (1µM), não foram observadas alterações (p>0,05), porém, nos tratamentos de 48 e 72 horas (p<0,05 em relação ao controle), houve diminuição da viabilidade ( Figura 1D ). Não houve diferença entre os tratamentos de 48 e 72 horas (p>0,05).

**Figura 2:** Efeitos de ICI, G-1 e rapamicina em células SKBr3. Viabilidade celular por MTT de células SKBr3 tratadas com ICI (A), G-1 (B) e rapamicina (C) por 24, 48 e 72 horas, em meio DMEM/F12, sem soro fetal bovino. Os dados representam a média de um experimento em triplicata. Valor de p<0,05* foi considerado significativamente diferente do controle, considerando desvio padrão da amostra. Nenhum dos compostos (ICI, G-1 e RAP) foi capaz de induzir a redução da viabilidade celular em SKBr3. Em D e E, marcação de células SKBr3 com laranja de acridina após tratamento com ICI (100nM), G-1 (100nM) e rapamicina (1µM) por 24 horas mostra que ICI e G-1 não induziram formação de compartimentos ácidos em SKBr3, linhagem que não expressa receptor de estrógeno alfa. Em D, gráficos de detecção da fluorescência em fluorescência verde e fluorescência vermelha. Em E, histograma mostrando valores médios (±erro médio padrão) em percentagem de células marcadas com laranja de acridina. Análise de variância de uma via, seguida do teste de Bonferroni (n=4). Grupo Controle, sem tratamento. Em E, *p<0,05 representa diferença estatística entre rapamicina *versus* Grupo Controle (sem tratamento) e G-1 CTR: Grupo Controle (sem tratamento); RAP: rapamicina; ICI: fulvestranto; LA: Laranja de acridina.

Na avaliação da viabilidade celular de células SKBr3 ( Figura 2A ), ao contrário do que ocorre nas células MCF-7, o ICI aumentou a viabilidade dependente do tempo e da concentração quando comparado ao Grupo Controle. Foi observado aumento de viabilidade celular nas concentrações de 50nM (48 horas), 100nM (24, 48 e 72 horas) e 200nM (24 e 48 horas). O tratamento com G-1 em células SKBr3 também aumentou a viabilidade celular em determinadas concentrações e tempo (na concentração de 1nM, em 24 horas, e nas concentrações de 10nM e 100nM, em 72 horas; Figura 2B ). A RAP não influenciou na viabilidade celular em células SKBr3, independente do tempo ( Figura 2C ). Para investigar se a ausência da autofagia, células SKBr3, que não expressaram REα, foram tratadas com ICI (100nM), G-1 (100nM) e RAP (1μM) por 24 horas ( Figura 2D e 2E ). As células foram marcadas com LA e analisadas por citometria de fluxo. Os dados demonstraram que ICI e G-1 não aumentaram o número de organelas vesiculares ácidas quando comparadas com o CTR (p>0,05) e RAP (p<0,05), sugerindo que, uma vez que não houve redução de viabilidade, a falta de autofagia pode justificar este resultado.

### Efeito do tratamento com ICI 182,780 e G-1 na indução de autofagia por meio da pontuação de GFP-LC3 e na formação de organelas vesiculares ácidas em células MCF-7

Durante a formação dos autofagossomos, ocorre a translocação específica de LC3 para a membrana do autofagossomo.^16^

Células MCF-7 expressando GFP-LC3 foram visualizadas por 4 horas após tratamento com ICI (100nM) e G-1 (100nM) ( Figura 3 ). Rapamicina (1μM) foi utilizada como controle positivo. Os resultados mostram que os tratamentos com ICI e G-1 não induziram translocação de GFP-LC3 que caracterizasse um aumento na autofagia quando comparados com o CTR. Por outro lado, a RAP aumentou a formação de autofagossomos, evidenciado pela translocação de GFP-LC3 e aumento de pontuações ( Figura 3A ).

**Figura 3:** Indução de autofagia induzida por rapamicina por meio da pontuação de GFP-LC3 em células MCF-7. Observa-se que ICI e G-1 não induziram autofagia no tempo observado (4 horas). Em A, imagens obtidas por microscopia de fluorescência de células MCF-7 expressando GFP-LC3 e estimuladas com rapamicina (1µM), ICI (100nM) e G-1 (100nM). Células foram mantidas em DMEM/F12 (CTR) e visualizadas a cada 30 minutos, durante 4 horas. (objetiva de 40x). Em B, histograma representando a quantificação de células que apresentaram aumento na pontuação de GFP-LC3 por 4 horas em células MCF-7. A média±erro médio padrão foi calculada a partir de cinco experimentos controles independentes. O limite superior foi obtido (média + duas vezes o erro médio padrão) e estabelecido como limite de corte, que, nesse caso, foi igual a um aumento de nove pontuações por célula. Para cada experimento, foi calculada a percentagem de células com aumento superior a esse limite. Análise de variância de uma via, seguida do teste de Bonferroni. Valores médios obtidos de pelo menos três experimentos independentes. Os grupos ICI, G-1 e rapamicina foram comparados ao Grupo Controle *p<0,05, representa diferença significante. CTR: Grupo Controle (sem tratamento); RAP: rapamicina; ICI: fulvestranto.

Conforme descrito anteriormente,^9^ a autofagia é um processo que pode ocorrer como forma de sobrevivência na presença de um estímulo estressante, avaliamos se esses efeitos estariam envolvidos na diminuição da viabilidade. Como os resultados demonstraram que não houve diminuição da proliferação em 24 horas, mas houve com 48 horas, prosseguiu-se com esses períodos de tratamento para investigar os efeitos de ICI e G-1 na formação de compartimentos ácidos pela marcação com LA e detecção por citometria de fluxo ( Figura 4 ). Os resultados mostraram que o tratamento com ICI (100nM) aumentou o número de compartimentos ácidos, tanto em 24 horas quanto em 48 horas, similar a RAP (1μM). Ambos os tratamentos foram significativamente diferentes (p<0,05) do CTR e não houve diferença entre os tratamentos com ICI e RAP (p>0,05), e entre os tratamentos de 24 e 48 horas ( Figura 4 ). Por outro lado, não foram observadas alterações nas medidas de compartimentos ácidos (p>0,05) nas células tratadas com G-1 ( Figura 4 ).

**Figura 4:** Efeito do ICI, G-1 e rapamicina sobre a formação de organelas vesiculares ácidas em células MCF-7. ICI e rapamicina induzem a formação de compartimentos ácidos em 24 e 48 horas em células MCF-7, enquanto G-1 não altera esse processo. Os histogramas apresentam valores médios±erro médio padrão em percentagem de células marcadas com laranja de acridina e analisadas por citometria de fluxo. Células MCF-7 foram tratadas com ICI (100nM), G-1 (100nM) e rapamicina (1µM) durante 24 horas (A e C) e 48 horas (B e D). Análise de variância de uma via, seguida do teste de Bonferroni. Em A, n=6; em B, n=5 * p<0,05 representa diferença significante entre ICI *versus* (Grupo Controle, sem tratamento, e G-1) rapamicina (Grupo Controle, sem tratamento, e G-1). CTR: Grupo Controle (sem tratamento); RAP: rapamicina; ICI: fulvestranto; LA: laranja de acridina.

### Efeito de 17β-estradiol e PPT na formação de compartimentos ácidos em células MCF-7 como indicativo de autofagia

Como o efeito de diminuição da viabilidade celular e do aumento de compartimentos ácidos em células MCF-7 ocorreu após o tratamento com ICI, mas não com G-1, esses efeitos poderiam estar associados com o antagonismo de REs e não envolveriam a ativação de GPER. Para investigar o efeito de agonistas de REs na autofagia, células MCF-7 foram tratadas por 24 horas com E2 (0,1nM, 1nM e 10nM) e comparadas com ICI (100nM). Os resultados mostraram que diferentes concentrações de E2 não foram capazes de aumentar os níveis de compartimentos ácidos nas células (p>0,05 comparado com CTR; Figura 5A e 5C ). Como E2 age nos diferentes RE, células MCF-7 foram tratadas por 24 horas com PPT (10nM e 100nM), um agonista específico de REα, que também não aumentou o nível de compartimentos ácidos (p>0,05 comparado ao controle) ( Figuras 5B e 5D ).

**Figura 5:** Detecção de organelas vesiculares ácidas por citometria de fluxo em células MCF-7 marcadas com laranja de acridina após tratamento com os compostos por 24 horas. A ativação dos receptores de estrógeno com 17β-estradiol, assim como a ativação seletiva de receptor de estrógeno alfa com PPT não induz formação de compartimentos ácidos em células MCF-7. Em A e C, células MCF-7 tratadas com 17β-estradiol nas concentrações 0,1nM, 1nM e 10nM. Em B e D, células MCF-7 tratadas com PPT nas concentrações de 10nM e 100nM. Em células MCF-7, os efeitos foram comparados com o ICI (100nM). Em A e B, gráficos de detecção da fluorescência em fluorescência verde e vermelha. Em C e D, os histogramas mostram valores médios±erro médio padrão em percentagem de células marcadas com laranja de acridina. Análise de variância de uma via, seguida do teste de Bonferroni (n=4). Em C e D, *p<0,05 representa diferença estatística em comparação com os outros grupos * p<0,05 representa diferença estatística em comparação com os outros grupos. CTR: Grupo Controle (sem tratamento); E2: 17β-estradiol; ICI: fulvestranto; LA: laranja de acridina.

## DISCUSSÃO

A autofagia é um mecanismo celular que pode levar à eliminação de componentes intracelulares quando da presença de um estímulo agressor ou pelo acúmulo de proteínas ou de organelas defeituosas.^9^ Alterações na via autofágica podem ocorrer em células cancerígenas e após quimioterapia. No câncer de mama, evidências apontam que proteínas envolvidas na via autofágica, como beclina-1, regulam o crescimento celular e interferem na sinalização de estrógeno.^18^

Estudos com os antagonistas de REs, como ICI e tamoxifeno, em células de câncer mamário, promovem aumento da autofagia^10 , 18 - 22^ que pode levar à resistência do câncer de mama e proliferação. Sabe-se que o ICI é antagonista de REα e REβ, mas foi relatado que, em modelos nos quais o ICI não tem efeito ou possui efeito agonista, sua ação deve ocorrer devido à ativação de GPER.^5^ Dessa forma, para o presente trabalho, compararam-se inicialmente as ações citotóxicas/antiproliferativas de ICI com G-1, um agonista seletivo de GPER. Verificou-se uma possível ação antiproliferativa de ICI em células MCF-7, que expressam REs, com tratamentos de 48 e 72 horas, pois não verificamos morte destas células nestas condições experimentais, em ensaios paralelos realizados (dados não mostrados). Sabe-se que o ICI causa uma diminuição da atividade de REα^23^ e seu efeito antiproliferativo está associado ao bloqueio dos REs clássicos, levando as células RE-positivas à morte celular por apoptose.^24^

Quanto ao envolvimento de GPER na proliferação celular, embora nossos resultados tenham demonstrado que a ativação seletiva de GPER pelo G-1 não tenha interferido nos parâmetros avaliados, trabalhos anteriores demonstraram os efeitos antiproliferativos e apoptóticos de G-1 em MCF-7, com concentração igual ou superior a 1µM de G-1, com pelo menos 72 horas de tratamento.^25 , 26^ Por outro lado, foi demonstrado que a via GPER pode estar envolvida no comportamento agressivo de tumores de mama, por meio da regulação da expressão da enzima aromatase, após inibição de GPER em células MCF-7 resistentes a tamoxifeno.^27^ Assim, GPER pode representar um alvo de tratamento no câncer de mama, e sua participação no crescimento de tumores de mama e na resistência tumoral após tratamento com antiestrógenos precisa ser melhor investigada.

Para verificar a participação dos RE e do GPER na autofagia, investigamos o efeito de ICI e G-1 na translocação de GFP-LC3, que é uma estimativa do processo de autofagia. Embora o ICI não tenha levado à translocação de GFP-LC3 neste estudo, isso pode estar relacionado com o tempo de exposição, que foi de 4 horas, e seria necessário um tempo maior para a indução da autofagia^10 , 28^ ou ainda poderia ocorrer a indução de vias autofágicas, que não envolvem a translocação de LC3.^7^ Já a ativação seletiva de GPER, com G-1, não induziu autofagia por translocação de LC3. Os efeitos de G-1 na autofagia ainda são desconhecidos e nas condições experimentais utilizadas no presente estudo, não foram observadas alterações significativas. Sobre a participação de GPER na autofagia, foi verificado que G-15, um antagonista de GPER, pode induzir autofagia independente de REs em linhagens de células de câncer de cabeça e pescoço humano, a despeito nos níveis de expressão de GPER.^29^ O papel de GPER na proliferação do câncer tem sido bastante explorado, porém a existência de uma interrelação entre esta via e a autofagia, um importante fenômeno de sobrevivência celular, não foi identificada.

Os experimentos com ICI em células MCF-7 mostraram aumento na marcação, com LA indicando maior número de compartimentos ácidos. Esse efeito não foi observado na linhagem SKBr3, que não expressa REα. Estudos demonstraram^10 , 28^ que o ICI pode levar à autofagia em células MCF-7 por outras metodologias, corroborando nossos resultados. Além de verificarem a translocação de LC3, checaram também a diminuição de p62, outro indicador da indução de autofagia. Essa proteína é requerida para a formação de agregados proteicos ubiquitinados, sendo direcionada e incorporada nos autofagossomos e, por fim, degradada nos autolisossomos.^16^ Sabe-se que o ICI causa uma diminuição da atividade de REα por induzir a degradação do receptor pelo sistema ubiquitina-proteassomo.^23^ A relação entre sistema ubiquitina-proteassomo com autofagia nos processos de captação de moléculas e degradação precisa ser mais bem investigada.^30^ Além disso, evidências apontam para a existência de uma interação de REα com beclina 1, uma das principais proteínas responsáveis pela formação de autofagossomos,^18^ sugerindo que a autofagia induzida por ICI tem relação principalmente com sua ação antagonista em REα.

Os resultados do MTT mostraram que a viabilidade de células SKBr3 não foi alterada com o tratamento com RAP 1µM por 24, 48 e 72 horas. De acordo com estudos clínicos, a RAP parece ter efeito citostático, pois a diminuição da proliferação do câncer de mama é modesta, mas quando utilizada em conjunto com outros quimioterápicos, o sinergismo é mais efetivo no tratamento.^31^ Apesar disso, a RAP induz formação de vesículas ácidas, que podem ser por indução de autofagia, embora não cause diminuição da viabilidade celular. Porém ressalta-se que para o presente estudo, a RAP foi utilizada como um controle positivo para verificar o aumento de organelas vesiculares ácidas e comparar com os efeitos de ICI e G-1.

Ao comparar os efeitos de ICI e G-1 em células SKBr3, alguns dos tratamentos, dependendo do tempo e da concentração, aumentaram a viabilidade celular dessas células, sem que fosse observada redução da viabilidade celular. Aparentemente, a resposta pela indução de GPER foi de sobrevivência em células RE-negativas. Paralelamente a esse evento, não ocorre estimulação na formação de vesículas ácidas, como lisossomos, que representam as últimas etapas da degradação de componentes macromoleculares e ocorrem na presença de estímulos causadores de estresse celular. Alguns estudos^11 , 32^ relataram efeitos pró-proliferativos de G-1 em células SKBr3 e também de ICI em células SKBr3, mostrando que a falta de expressão de REs, principalmente REα, e a presença de GPER podem determinar efeitos proliferativos em células de câncer de mama negativas para receptores de estrógeno, demonstrando porque os tratamentos com antiestrógenos, como o tamoxifeno e ICI, não são efetivos nesse tipo de câncer de mama.

Depois que foi estabelecida a relação entre autofagia e câncer, diversas pesquisas foram realizadas, a fim de descobrir o papel da autofagia e sua relação com o câncer e resposta terapêutica, avaliando a relação entre a via autofágica e a proliferação e/ou morte celular. Em relação ao câncer de mama, estudos verificaram que antagonistas de REs, como ICI e tamoxifeno, em células de câncer mamário, promoveram o aumento da autofagia.^10 , 18 - 22^ Evidências recentes mostraram que a inibição da autofagia é capaz de induzir morte celular, sendo a autofagia um mecanismo de sobrevivência,^22 , 33 , 34^ e, no caso de células de câncer de mama positivas para receptores de estrógeno, a autofagia induzida pelo ICI parece contribuir para a resistência do câncer.^10^ Como ICI tem efeito de ativação em GPER, ainda não foi investigado se a via GPER está envolvida com a sobrevivência pelo processo autofágico. Assim, é importante avaliar qual o papel desse receptor nos mecanismos de sobrevivência do câncer mamário, tornando-se um importante alvo no tratamento do câncer.

## CONCLUSÃO

Um problema importante na terapia do câncer de mama é a resistência adquirida após tratamento com antiestrógenos, como o ICI. Um de seus alvos, o receptor de estrógeno acoplado a proteína-G, parece ter papel importante na proliferação/viabilidade de células cancerígenas, e sua ação parece variar dependendo das condições do meio. Um mecanismo celular de proteção bastante investigado atualmente é a ativação da autofagia. O efeito do ICI no aumento de compartimentos ácidos em células positivas para receptores de estrógeno parece estar associado principalmente com seu efeito de inibição dos receptores de estrógenos, e o receptor de estrógeno acoplado a proteína-G parece não estar envolvido. Esses dados podem ser importantes para compreender os mecanismos de morte celular e resistência, visando a novas alternativas terapêuticas.
